# Prevalence of fatigue at one-year follow-up from the Gothenburg recovery and rehabilitation after COVID-19 and intensive care unit study

**DOI:** 10.1038/s41598-022-14787-6

**Published:** 2022-07-12

**Authors:** Netha Hussain, Carina M. Samuelsson, Avril Drummond, Carina U. Persson

**Affiliations:** 1 Department of Radiology, Sahlgrenska University Hospital, Gothenburg, Region Västra Götaland Sweden; 2 Department of Occupational Therapy and Physiotherapy, Sahlgrenska University Hospital/Östra, Gothenburg, Region Västra Götaland Sweden; 3 Faculty of Medicine and Health Sciences, University of Nottingham, Nottingham, UK; 4 Department of Clinical Neuroscience, Rehabilitation Medicine, Institute of Neuroscience and Physiology, Sahlgrenska Academy, University of Gothenburg, Gothenburg, Sweden

**Keywords:** Risk factors, Infectious diseases

## Abstract

Fatigue was a commonly reported sequala after COVID-19. However, there is little literature about the prevalence and predictors of fatigue one year after Intensive Care Unit (ICU) admission following COVID-19. Therefore, the aim of this study was to determine the prevalence of fatigue and to identify the predictors prior to, and during the care period in ICU that were associated with fatigue at one year after ICU admission following COVID-19. The dependent variable, fatigue, was assessed using the Swedish version of Fatigue Assessment Scale (S-FAS), in a cohort of 105 individuals cared for at the ICU at the Sahlgrenska University hospital, Sweden during the first wave of the pandemic. The independent variables were related to demographic factors, comorbidities and complications during ICU admission following COVID-19. Fatigue was reported by 64.4% (n = 67) of the individuals. Age (odds ratio: 0.95, confidence interval: 0.92–0.99) and length of stay in the ICU (odds ratio: 1.04, confidence interval: 1.00–1.07) were statistically significant predictors of fatigue one year after ICU admission following COVID-19. The findings from this study will be important for healthcare practitioners, policy makers and the general public in planning the rehabilitation of individuals who underwent ICU care for COVID-19.

## Introduction

A wide variety of symptoms and sequala are reported after COVID-19^1^, of which the most common symptom is fatigue^2,3^. Fatigue is classified into the domain of body functions according to the International Classification of Functioning, Disability and Health (ICF)^4^. Post-COVID-19 is referred to as “the decrease in physical and/or mental performance that results from changes in central, psychological, and/or peripheral factors due to the COVID-19 disease”^5^. Previous literature shows that the prevalence of fatigue was 52% at 10 weeks^6^, 13–47% at 16–20 weeks^7,8^ and 10–35% at 6 months^7^ after COVID-19. The prevalence of post-COVID-19 fatigue was much higher in hospitalized individuals, with 36–53% reporting fatigue soon after the recovery from acute symptoms^9,10^, 26% at 3 months^11^, 63% at 6 months^12^ and 20% at 1 year^13^ after discharge from the hospital. In focus group interviews, individuals have frequently reported difficulty in performing daily life tasks due to post-COVID-19 fatigue^14^. Post-COVID-19 sequala, such as fatigue, are important from a public health perspective because they can lead to decrease in quality of life and societal participation^15^.

In Sweden, approximately 1.8% of those who tested positive for COVID-19^16^, and 16.2% of those hospitalized for COVID-19, required care in an Intensive Care Unit (ICU)^17^. In individuals requiring ICU care, the prevalence of fatigue was 72% at 4–8 weeks^18^, while up to 16% reported fatigue at 3–6 months after ICU admission^19^. When individuals admitted to the ICU were compared with individuals admitted to a ward due to COVID-19, no differences were found in terms of fatigue 11 days after COVID-19^15^. However at 4–8 weeks after discharge from hospital, higher level of fatigue was seen in individuals with COVID-19 admitted to the ICU^18^. At 3 months after admission to the ICU for COVID-19, 88% had not regained their baseline level of activity despite receiving inpatient rehabilitation^20^. At one year after COVID-19, fatigue was reported by 20% of those who required supplemental oxygen and 22% of those requiring invasive or non-invasive oxygen support for COVID-19^13^. Nevertheless, at one year after ICU admission for COVID-19, it is clear that there is a dearth of data concerning the prevalence of fatigue^21^.

A few studies have examined the factors related to fatigue in individuals who were hospitalized after COVID-19^6,11,22^, including those who required ICU care^23^. From 6 weeks up to 6 months after hospitalization for COVID-19, females were found to be more likely to experience fatigue^6,11,22^. At 3 months after COVID-19, the duration of stay in the hospital was longer for those who had fatigue compared with those who did not^11^. At follow-up during 6 months after diagnosis of COVID-19, no association was found between the presence of fatigue and the severity of acute COVID-19^23^. However, there is little literature about the predictors of fatigue at one year after admission to ICU following COVID-19. Therefore, the aim of this study was to determine the prevalence of fatigue in this cohort and to identify the predictors prior to and during the care period in ICU, that are associated with fatigue at 1 year after ICU admission following COVID-19. Our hypothesis was that fatigue at 1 year after ICU admission for COVID-19 was associated particularly with sex and length of stay at the ICU, along with co-morbidities and complications that arose during ICU stay.

## Methods

### Study design

The participants of this study were recruited from the Gothenburg Recovery and Rehabilitation after COVID-19 and Intensive Care Unit (GOT-RECOV-19 ICU) study. GOT-RECOV-19 ICU is a multicenter cohort study, in which outcome measures were collected retrospectively at one year after ICU admission following COVID-19. In the current study, the sections concerning prevalence and predictors use cross-sectional and longitudinal observational designs, respectively. The GOT-RECOV-19 ICU was registered at “FoU i Sverige” (researchweb.org), the open project database for research projects from Sweden, on May 28, 2020 (ID number: 274477). All methods were performed in accordance with the relevant guidelines and regulations of the Swedish Ethical Review Authority.

### Inclusion and exclusion criteria

All adult individuals (defined as 18 years or above) admitted to any of the five Intensive Care Units (ICUs) treating patients with COVID-19 at Sahlgrenska University Hospital (SU) between 2020-03-01 and 2020-06-30, with the diagnostic code UO7.1 (COVID-19 virus detected, according to classification system ICD-10 SE) and still alive one year after admission to the ICU, met the study criteria. The exclusion criteria were: individuals not registered as a resident of Sweden in the Swedish Population Register or not living in Gothenburg or its surrounding municipalities.

### Procedures

**Figure 1:**
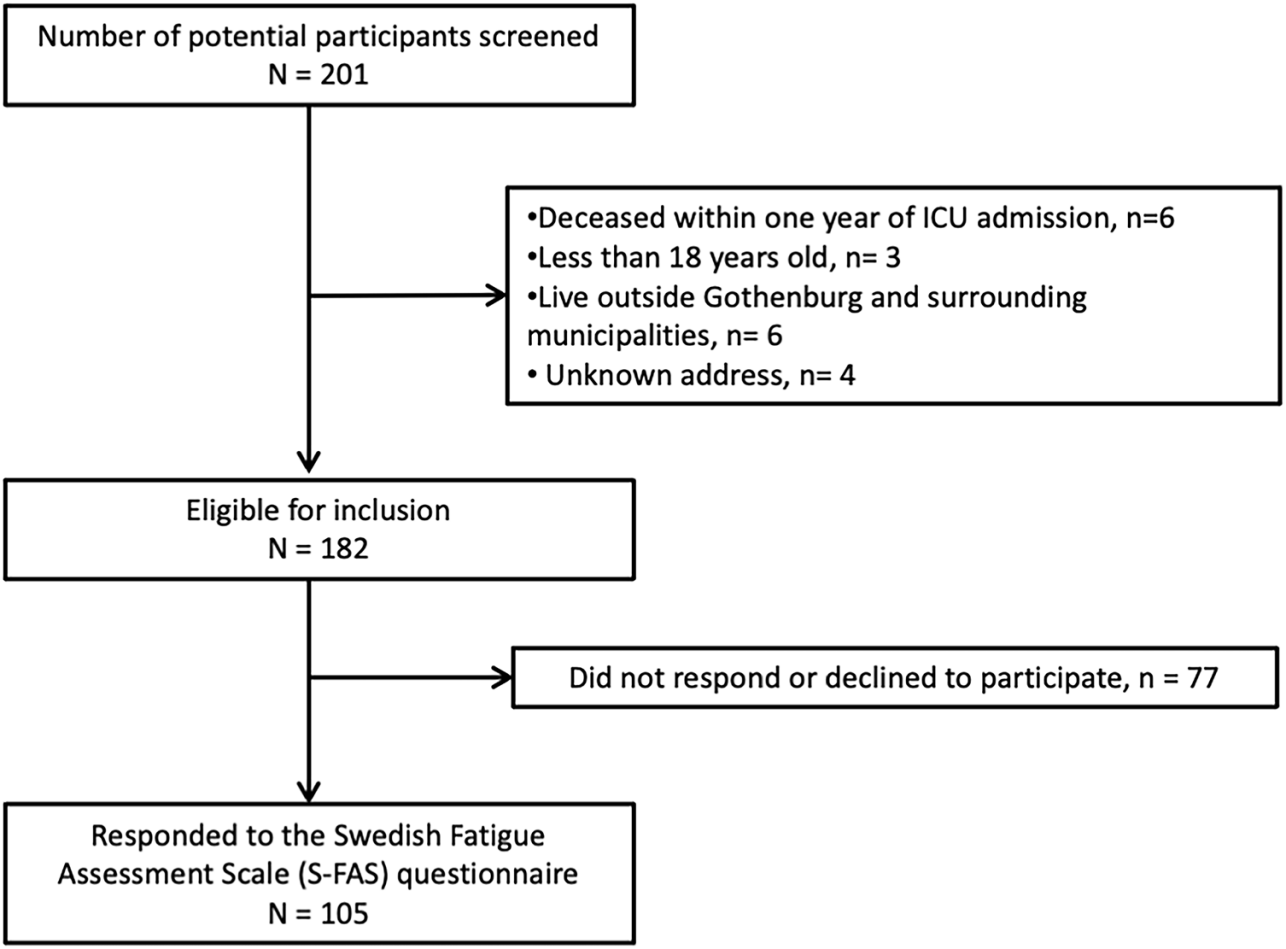
Flowchart of the inclusion process.

In March 2021, the patient record data for those admitted to the ICU with COVID-19 as the primary diagnosis was collected from the Output Unit at Sahlgrenska University Hospital. Two hundred and fifty-nine patients who received care in the ICU after COVID-19 were identified, out of which 58 had died before the one-year follow-up. After a review of the 201 surviving individuals, 19 were excluded as they did not meet the inclusion criteria (Fig. 1). The remaining 182 potential participants were sent a postal invitation/request for study participation, including clinical assessments and questionnaires. This invitation was followed by up to two reminders if they did not respond to the first invitation. In the second reminder, the participants were given an option to respond exclusively to the questionnaires and send them back postally or answer digitally using a link, without completing the clinical assessments.

### Assessment of independent variables at baseline

For those who consented to participate in the study, the following independent variables were obtained from the medical records: age, sex and presence of comorbidities (hypertension; diabetes mellitus; chronic kidney disease; coronary heart disease; chronic heart failure; chronic obstructive pulmonary disease and asthma). Determination of presence of comorbidities was based on the consulting doctor’s assessment of the individual on arrival at the hospital. Additional independent variables were the presence of complications such as sepsis, Acute Respiratory Distress Syndrome (ARDS) and embolic events (such as myocardial infarction, ischemic stroke, pulmonary embolism, deep vein thrombosis) (yes/no) during the care period in the ICU.

### Primary outcomes

The data collection of the primary outcomes: fatigue, physical fatigue and mental fatigue started in March 2021 and was performed consecutively at one year after the admission to the ICU following COVID-19. Fatigue was assessed using the Swedish language version (S-FAS)^24^ of the Fatigue Assessment Scale (FAS). The FAS is a self-reported, 10-item ordinal questionnaire with five response categories per item (1 to 5), and a total score ranging from 10 to 50^25,26^. Higher scores indicate greater fatigue. Items 1–2, 4–5 and 10 of the FAS assess physical fatigue, while items 3 and 6–9 assess mental fatigue. Items 4 and 10 require reversed scoring. A total FAS score ≥ 22 indicates the presence of fatigue^27^. The cut-off scores for both physical and mental fatigue were considered to be ≥ 11, although FAS has not been validated for sub-scores. The FAS has good reliability and validity in healthy workers^25,28^, individuals with stroke^29^ and in individuals with chronic heart failure^29^. Furthermore, the S-FAS has been tested for reliability and validity in individuals with stroke^24^. As there are no validation studies examining the psychometric properties of S-FAS in individuals with COVID-19, the cut-off value of ≥ 22 was used based on previous observational studies examining fatigue in individuals with sarcoidosis^27^ and COVID-19^2^.

All assessments for the GOT-RECOV-19 ICU were conducted at the Sahlgrenska University Hospital/Östra and all data collection for the baseline and outcome variables was performed independently by two physiotherapy researchers (CUP, CMS). For those who underwent clinical assessments at the hospital, several outcome measures corresponding to diverse domains of the International Classification of Functioning and Health (ICF)^4^ were assessed. The assessment of fatigue was performed as the sixth outcome in the order of the battery of assessments for the GOT-RECOV-19 ICU study, taking into consideration that the responses to earlier assessments could unintentionally influence the responses to the subsequent assessments, leading to biased responses.

For encouraging public participation and partnership in determining the most important research areas surrounding post-COVID-19, we contacted the Swedish COVID association (https://covidforeningen.se/ a registered body that aims to increase awareness about COVID-19 and its sequelae). Upon our request, they expressed interest in ranking the COVID-19 related symptoms and sequelae being investigated as a part of GOT-RECOV-19 ICU in the order of importance. In October 2021, the members of the association were asked to voluntarily answer an email questionnaire via their official website and social media. Fatigue was ranked as the most important sequela by the members, which was why it was chosen as the primary outcome for the current study, which is the first one from the GOT-RECOV-19 ICU cohort.

### Statistical analyses

Based on data from previous studies, the lowest prevalence of post-COVID-19 fatigue was 40%^12,21^, compared to approximately 20%^30^ fatigue in the general population. Using this assumption and expecting a drop-out rate of 40%, we aimed to include at least 105 individuals for obtaining a power of 80% and a margin of error of 0.05 (two-tailed).

The statistical analyses were performed using the IBM Statistical Package for Social Sciences (SPSS) software, version 28. The level of significance was set to p < 0.05 (two-tailed). The comparison of the demographic characteristics between those who participated in the study and those who declined to participate was performed using the Mann–Whitney U-test. A sub-analysis using Chi square test was performed to identify whether there was any significant difference in fatigue between those who responded during different seasons^31^ of the year.

Descriptive statistics, such as means, standard deviations (SDs), absolute numbers, percentages, medians and interquartile ranges (IQRs) were presented, showing the prevalence of comorbidities and complications. The results of S-FAS scoring were also presented. Binary logistic regression analysis was used to identify the factors that were associated with the three dependent variables: fatigue (using the total S-FAS score), physical fatigue (using the S-FAS physical score) and mental fatigue (using the S-FAS mental score) at one year after ICU care. Each of the three dependent variables was dichotomized into two groups based on the presence of fatigue, physical fatigue and mental fatigue (yes/no). For the three participants who missed to answer one item each in the S-FAS questionnaire, interval estimation was performed to calculate the dichotomized values.

Multicollinearity between the independent variables was determined using Spearman’s rank correlation, where correlation coefficients of ≥ 0.7 were considered as multicollinear^32^. Univariable logistic regression was performed first, after which variables with *p*-values of < 0.1 were included in the multivariable logistic regression model. In the multivariable regression model, a *p*-value of < 0.05 was considered to be statistically significant.

The goodness-of-fit for the multivariable logistic model was tested using the Hosmer–Lemeshow test and the improvement in fit was verified using Cox & Snell and Nagelkerke pseudo R^2^ values. The results of the logistic regression models were presented as odds ratios (ORs), 95% confidence intervals (CIs) and *p*-values. The area under the receiver operating characteristic (ROC) curve was calculated for the final prediction model. An area of 70–79% under the curve was regarded as acceptable, 80–89% was regarded as excellent and 90–100% was regarded as outstanding diagnostic accuracy^33^.

### Ethical declarations

Informed written consent was obtained from all participants prior to their inclusion in the study. Ethical approval was obtained on September 24, 2020 (ID number: 2020-03264) from the Swedish Ethical Review Authority, which also approved the sending of the reminder mails to the participants on June 29, 2021 (ID number: 2021-03220).

## Results

Of the 182 participants who met the inclusion criteria, 105 (57.7%) responded to the study questionnaire. Of these, 78 participants responded to the questionnaire during their visit to the hospital, 26 responded postally and one responded digitally. Between the 105 individuals who participated in the study and the 79 individuals who declined to participate/did not respond to the study request, we found no significant differences in terms of age, sex, or length of stay at the ICU. No statistically significant difference in the presence of fatigue was found in the participants who responded in spring or summer.

The demographic characteristics and scores of the study group at the baseline are presented in Table 1. Seventy-two participants responded to the questionnaire within 14 days after completion of one year of ICU admission and 33 participants responded after 14 days, at median 59 (IQR: 25–84) days after the set time frame. Out of them, 29 were those who responded to the second reminder invitation. Most of the participants were male and younger than 65 years of age. Of the total 54 individuals whose BMI was available, close to three-fourth were either obese or overweight. Six individuals (5.7%) experienced an embolic event during ICU care. One in two individuals had sepsis (52%) or ARDS (53%), while one in three had both (35%). Three individuals (2.9%) required extracorporeal membrane oxygenation (ECMO) therapy.

**Table 1:** Demographic characteristics for the 105 participants at baseline.

Characteristics	All participants [Mean ± SD, Median (IQR), n (%)]
Age (years)	58.18 ± 11.83
Age over 65 years	29 (27.6)
Female	25 (23.8)
Male	80 (76.2)
Height (cm) (n = 68)	173.7 ± 16.7
Weight (kg) (n = 65)	94.1 ± 20.2
BMI (kg/m^2^) (n = 54)	29.7 ± 7.9
Length of stay in ICU (days)	15 (7.5–26.5)
**Type of respiratory support**	
Mask or nasal cannula	4 (3.8)
High Flow Oxygen Therapy	15 (14.3)
Mechanical ventilation (i.e., intubated)	86 (81.9)
**Employment status at ICU admission**	
Employed	75 (71.4)
Unemployed	2 (1.9)
Sick leave, part time	1 (1.0)
Sick leave, full time	3 (2.9)
Early retired	3 (2.9)
Retired	21 (20.0)
**Comorbidities**	
Hypertension	44 (42.0)
Coronary heart disease	27 (26.7)
Diabetes mellitus	23 (21.9)
Asthma	8 (7.6)
Chronic kidney disease	6 (5.7)
Chronic heart failure	4 (3.8)
Chronic obstructive pulmonary disease	2 (1.9)

*SD* standard deviation, *IQR* interquartile range, *BMI* body mass index.

**Figure 2:**
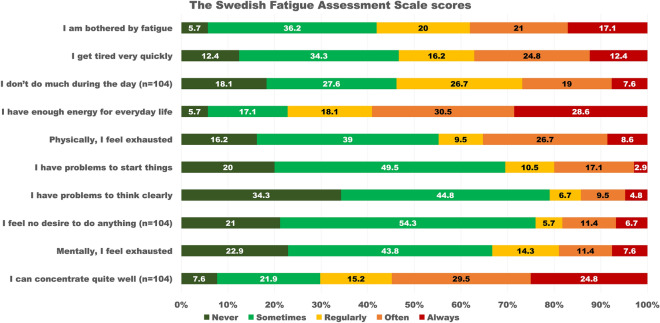
Proportions of the Swedish Fatigue Assessment Scale (S-FAS) scores of the study population. The original scores are presented also for items 4 and 10.

Results from the S-FAS questionnaire are shown in Fig. 2 and Table 2. These results showed that over 1 in 5 individuals never or sometimes had enough energy to manage daily life. One in 5 individuals often or always had trouble in starting things. Approximately 1 in 5 individuals reported having mental exhaustion often or always (Fig. 2). The results also showed that nearly two in three individuals experienced fatigue. More than two thirds experienced physical fatigue and more than one-half experienced mental fatigue at one year after ICU-admission following COVID-19 (Table 2).

**Table 2:** The dependent variable, Swedish Fatigue Assessment Scale (S-FAS), at one year after ICU admission following COVID-19.

S-FAS at one year (N = 105)	Median (IQR) score/n (%)
S-FAS total score (n = 102)	25 (19–31)
S-FAS physical score (n = 104)	13 (9–18)
S-FAS mental score (n = 103)	11 (9–14)
Fatigue (i.e., a S-FAS total score ≥ 22) (n = 104)	67 (64.4)
Physical fatigue (i.e., a S-FAS physical score ≥ 11)	70 (67.3)
Mental fatigue (i.e., a S-FAS mental score ≥ 11)	58 (56.3)

*IQR* interquartile range, *S-FAS* the Swedish Fatigue Assessment Scale (FAS) (range 10–50), *S-FAS physical score* the five S-FAS items that relate to physical fatigue (range 5–25), *S-FAS mental score* the five S-FAS items that relate to mental fatigue (range 5–25).

No multicollinearity was found between the independent variables. The results of the univariable analysis are shown in Table 3. Age had a significant negative association and length of stay at the ICU had a significant positive association with fatigue at 1 year after ICU admission following COVID-19. Similarly, increased length of stay at the ICU was significantly associated with physical fatigue and age was significantly negatively associated with mental fatigue at 1 year after ICU care for COVID-19. Other independent variables such as comorbidities and complications did not show significant associations with fatigue.

**Table 3:** Univariable analyses for prediction of fatigue in ICU-admitted individuals at one year after COVID-19.

Predictor	Univariable analysis	
	Odds ratio (95% CI)	*P*-value
Dependent variable: A total score ≥ 22 of the Swedish Fatigue Assessment Scale (n = 104*)		
Age	**0.96 (0.92–0.99)**	**0.042**
Female sex (male sex = ref.)	1.54 (0.58–4.21)	0.37
Length of stay in ICU	**1.04 (1.00–1.07)**	**0.032**
Hypertension	1.12 (0.50–2.53)	0.79
Diabetes mellitus	1.76 (0.63–4.94)	0.29
Coronary heart disease	1.06 (0.42–2.68)	0.91
Asthma	4.20 (0.50–35.54)	0.19
Sepsis	0.93 (0.42–2.08)	0.85
Embolic event	2.90 (0.33–25.84)	0.34
ARDS	0.90 (0.75–1.08)	0.26
**Dependent variable: A physical fatigue score of ≥ 11 of the Swedish Fatigue Assessment Scale (n = 105)**		
Age	0.99 (0.95–1.02)	0.42
Female sex (male sex = ref.)	1.34 (0.50–3.60)	0.57
Length of stay in ICU	**1.06 (1.02–1.11)**	**0.006**
Hypertension	1.87 (0.79–4.40)	0.15
Diabetes mellitus	1.50 (0.53–4.22)	0.45
Coronary heart disease	2.00 (0.72–5.54)	0.18
Asthma	3.31 (0.36–26.78)	0.31
Sepsis	0.59 (0.25–1.35)	0.21
ARDS	0.85 (0.71–1.03)	0.10
Embolic event	2.54 (0.29–22.63)	0.41
Dependent variable: A mental fatigue score of ≥ 11 of the Swedish Fatigue Assessment Scale (n = 104*)		
Age	**0.96 (0.92–0.99)**	**0.02**
Female sex (male sex = ref.)	1.92 (0.74–4.96)	0.18
Length of stay in ICU	0.98 (0.96–1.00)	0.10
Hypertension	0.75 (0.34–1.65)	0.48
Diabetes mellitus	2.07 (0.77–5.57)	0.15
Coronary heart disease	1.33 (0.54–3.31)	0.54
Asthma	1.32 (0.30–5.85)	0.71
Sepsis	1.18 (0.54–2.57)	0.69
ARDS	0.92 (0.77–1.10)	0.36
Embolic event	1.59 (0.28–9.11)	0.60

*CI* confidence interval, *ICU *intensive care unit, *ARDS* acute respiratory distress syndrome.

*Interval estimation failed to calculate the dichotomized S-FAS total score for one of the 105 participants.

Significant values are given in bold.

Multivariable logistic regression analysis was performed with age [OR: 0.95 (0.92–0.99), *p* = 0.018] and length of stay in the ICU [OR: 1.04 (1.00–1.07), *p* = 0.018] as independent variables and fatigue as the dependent variable. The accuracy of this model was estimated to be 70.4% using the area under the ROC curve, showing that it has acceptable accuracy. The significance of Hosmer–Lemeshow test was 0.05, Cox & Snell R Square was 0.12 and Nagelkerke pseudo R Square was 0.16.

For the dependent variables physical fatigue and mental fatigue, only one independent variable each was found to be significant for inclusion in the final model. Therefore, it was not appropriate to perform any multivariable regression analysis for them.

## Discussion

In this follow-up study, we found that nearly two-thirds of the individuals reported fatigue at one year after ICU admission following COVID-19. Our hypothesis that increased length of stay in the ICU, a proxy for COVID-19 severity, was a predictor of fatigue was confirmed. However, our hypothesis that sex, comorbidities and complications after the ICU stay were predictors of fatigue at one year follow-up was rejected.

The proportion of individuals with severe COVID-19 who reported fatigue at a one-year follow-up is higher in the current study than in a related study where 20–22% of the individuals reported fatigue and/or muscle weakness using a symptom questionnaire^13^. It is likely that the assessment performed in the current study allowed for a more extensive evaluation of fatigue using the S-FAS, which is why higher prevalence of fatigue is observed. Another study reported fatigue in 30% individuals at one year after hospitalization for COVID-19^10^. Our study only included ICU-admitted individuals who are likely to have had more severe COVID-19, which could explain the differences in results.

Our finding that length of stay in the ICU was positively associated with fatigue at 1 year in the current study is in keeping with previous research which assessed fatigue at 3 months after hospitalization for COVID-19^11^. In contrast, at 6 months follow-up after COVID-19, the severity of the disease was not found to be a significant predictor of fatigue^23^. The current study used the length of ICU stay and complications during ICU admission as the indicator for severity of COVID-19, while the 6-month follow-up study measured the infection severity using a combination of indicators, including length of hospital stay.

Interestingly, we found no significant association between higher age and female sex with fatigue at one year after ICU admission for COVID-19. It is probable that healthier and younger individuals notice a larger decline in their health after COVID-19, compared to the older individuals who might already been experiencing age-related aliments and tiredness, and therefore self-report more fatigue than their older counterparts. Additionally, FAS, the assessment scale used in this study is known to be not sufficiently sensitive for measuring age differences in relation to fatigue^25^. Contrary to the findings from past studies at 8 months and one year following COVID-19^13,34^, sex was not found to be a predictor of fatigue in the current study. Since male sex is a risk factor for ICU admission for COVID-19^35^, we had fewer women in our cohort (24%), and this low proportion of women could be a reason why significant differences could not be demonstrated in our study. Furthermore, we could not test the hypothesis that high BMI was a predictor for post-COVID-19 fatigue at one year due to missing data related to weight and height for nearly half of the participants.

To our knowledge, this is the first study in which the predictors of fatigue at one year after ICU admission for COVID-19 have been identified. The strength of this study is the consecutive inclusion of participants from a relatively large and well-defined geographical area in Sweden with those affected in the first wave of the pandemic.

This study is not without limitations. The S-FAS questionnaire, which is a Patient Reported Outcome Measure (PROM), used in this study could be quickly and easily administered to the participants. However, S-FAS has not been validated in individuals with COVID-19, which may be considered a limitation of this study. Some individuals who declined to participate in the GOT-RECOV-19 ICU cohort responded that they have already been involved in several other research projects surrounding COVID-19, which could be why they did not respond to the participation request or declined to participate in the current study. As in other prediction studies, important variables that are likely to predict fatigue at one year after ICU admission following COVID-19 may have not been included in the current study. Fatigue experienced due to psychological reasons, such as due to pandemic restrictions in social participation and presence of newly diagnosed diseases within the 1-year period after ICU admission were not included in the scope of this study, which may have affected the results. The study design was unable to show whether fatigue was caused by COVID-19 per se, or by the ICU stay, or by a combination of both*.* However, of note, in a pre-COVID-19 study of 89 individuals who required ICU admission due to conditions other than trauma, the prevalence of fatigue assessed using the Lee Fatigue Scale was found to be 13.8%^36^. Although 42% of the eligible individuals did not participate in the study, there were no significant demographic differences between the participants and the non-participants, indicating that the study results are generalizable. However, the well-defined nature of the population limits the generalizability of the study results to individuals cared for at the ICU for COVID-19.

## Conclusion

The prevalence of fatigue at one year after ICU admission for COVID-19 was high and those who had a longer duration of ICU care were particularly vulnerable to fatigue. The predictors identified in this study, younger age and longer stay at the ICU, are important factors for healthcare practitioners, policy makers and the general public to consider in planning rehabilitation care for individuals who underwent ICU care for COVID-19.

## Data Availability

The dataset is available from the principal investigator, Carina U. Persson (carina.persson@vgregion.se), in response to a reasonable request. According to Swedish regulations, permission to use data can be obtained after an application to and approval by the Swedish Ethical Review Authority.
