# Author Correction: Prevalence of fatigue at one-year follow-up from the Gothenburg recovery and rehabilitation after COVID-19 and intensive care unit study

**DOI:** 10.1038/s41598-023-39074-w

**Published:** 2023-07-24

**Authors:** Netha Hussain, Carina M. Samuelsson, Avril Drummond, Carina U. Persson

**Affiliations:** 1 Department of Radiology, Sahlgrenska University Hospital, Gothenburg, Region Västra Götaland Sweden; 2 Department of Occupational Therapy and Physiotherapy, Sahlgrenska University Hospital/Östra, Gothenburg, Region Västra Götaland Sweden; 3 Faculty of Medicine and Health Sciences, University of Nottingham, Nottingham, UK; 4 Department of Clinical Neuroscience, Rehabilitation Medicine, Institute of Neuroscience and Physiology, Sahlgrenska Academy, University of Gothenburg, Gothenburg, Sweden

Correction to: *Scientific Reports* 10.1038/s41598-022-14787-6, published online 12 July 2022

The original version of this Article contained errors in Table 1, where the “All participants” values for “High Flow Oxygen Therapy” and “Mechanical ventilation (i.e., intubated)” under “Type of respiratory support” were incorrect. The correct and incorrect values appear below.

Incorrect:

**Table:** 

Characteristics	All participants [Mean ± SD, Median (IQR), n (%)]
**Type of respiratory support**	
High Flow Oxygen Therapy	13 (12.4)
Mechanical ventilation (i.e., intubated)	88 (83.8)

Correct:

**Table:** 

Characteristics	All participants [Mean ± SD, Median (IQR), n (%)]
**Type of respiratory support**	
High Flow Oxygen Therapy	15 (14.3)
Mechanical ventilation (i.e., intubated)	86 (81.9)

The original Article has been corrected.

